# A Deep Learning Workflow for Mass-Forming Intrahepatic Cholangiocarcinoma and Hepatocellular Carcinoma Classification Based on MRI

**DOI:** 10.3390/curroncol30010042

**Published:** 2022-12-30

**Authors:** Yangling Liu, Bin Wang, Xiao Mo, Kang Tang, Jianfeng He, Jingang Hao

**Affiliations:** 1 Faculty of Information Engineering and Automation, Kunming University of Science and Technology, Kunming 650504, China; 2 Department of Radiology, Second Affiliated Hospital of Kunming Medical University, Kunming 650101, China

**Keywords:** mass-forming intrahepatic cholangiocarcinoma, hepatocellular carcinoma, liver cancer classification, deep learning, residual network

## Abstract

Objective: Precise classification of mass-forming intrahepatic cholangiocarcinoma (MF-ICC) and hepatocellular carcinoma (HCC) based on magnetic resonance imaging (MRI) is crucial for personalized treatment strategy. The purpose of the present study was to differentiate MF-ICC from HCC applying a novel deep-learning-based workflow with stronger feature extraction ability and fusion capability to improve the classification performance of deep learning on small datasets. Methods: To retain more effective lesion features, we propose a preprocessing method called semi-segmented preprocessing (Semi-SP) to select the region of interest (ROI). Then, the ROIs were sent to the strided feature fusion residual network (SFFNet) for training and classification. The SFFNet model is composed of three parts: the multilayer feature fusion module (MFF) was proposed to extract discriminative features of MF-ICC/HCC and integrate features of different levels; a new stationary residual block (SRB) was proposed to solve the problem of information loss and network instability during training; the attention mechanism convolutional block attention module (CBAM) was adopted in the middle layer of the network to extract the correlation of multi-spatial feature information, so as to filter the irrelevant feature information in pixels. Results: The SFFNet model achieved an overall accuracy of 92.26% and an AUC of 0.9680, with high sensitivity (86.21%) and specificity (94.70%) for MF-ICC. Conclusion: In this paper, we proposed a specifically designed Semi-SP method and SFFNet model to differentiate MF-ICC from HCC. This workflow achieves good MF-ICC/HCC classification performance due to stronger feature extraction and fusion capabilities, which provide complementary information for personalized treatment strategy.

## 1. Introduction

Intrahepatic cholangiocarcinoma (ICC) is a primary malignant tumor of the liver. It can be classified into mass-forming, periductal-infiltrating, intraductal-growth, and mixed types based on growth pattern. Among them, mass-forming intrahepatic cholangiocarcinoma (MF-ICC) accounts for about sixty percent of ICC; it is the second most common primary liver malignancy following hepatocellular carcinoma (HCC) [1]. MF-ICC and HCC share similar risk factors, including cirrhosis and chronic viral hepatitis. However, these two tumors have distinctly different treatment strategies. Surgical removal of the tumor affords the only chance of a cure for MF-ICC, while percutaneous ablation, radiofrequency ablation, surgical resection, and liver transplantation are available options for HCC [2,3,4,5]. Therefore, accurate preoperative differentiation of MF-ICC from HCC has great clinical importance.

Some current studies attempted to identify new quantitative biomarkers for MF-ICC/HCC classification. Zou et al. found that the MF-ICC and HCC had significant differences in volumetric apparent diffusion coefficient histogram parameters [6]. Zhao et al. indicated that liver perfusion parameters and corresponding histogram parameters also provided useful value in differentiating MF-ICC from HCC [7]. Wu et al. used multivariate analysis to determine the strongest predictors distinguishing MF-ICC from HCC [8]. Zheng et al. applied a multivariable logistic regression analysis to find reliable predictors for MF-ICC/HCC classification; the results showed that enhancing the “capsule” was a reliable imaging feature to distinguish the two types of tumors [9]. The aforementioned studies applied traditional method, the main steps including lesion delineation, feature extraction, and statistical analysis. However, these processes were subjective, laborious, and time-consuming, thus limiting their clinical applicability. In contrast, deep learning extracts relevant high-level features automatically from the raw images, not only avoiding manual feature extraction, but also performing better than other machine learning methods [10,11,12].

Deep learning algorithms are widely used in the field of medical imaging, and they have also achieved good results in the classification of liver tumors. Zhen et al. used a convolutional-neural-network (CNN)-based method to classify liver tumors on MRI; the results showed that the deep learning model achieved a performance on par with experienced radiologists in classifying liver tumors in seven categories [13]. Oestmann et al. constructed a 3D CNN to classify multiphase T1WI MRI of HCC and non-HCC; the model achieved an accuracy of 87.3% and area under the receiver operating curve (AUC) of 0.912 [14]. However, these studies have only treated ICC as a whole and did not consider MF-ICC as a separate category.

In clinical practice, HCC and MF-ICC have a different enhancement pattern on contrast-enhanced computed tomography (CT) or MRI [15,16]. HCC exhibits homogeneous or heterogeneous hyperenhancement in the arterial phase, followed by washout during the dynamic. Conversely, MF-ICC often presents with peripheral enhancement or heterogeneous hypoenhancement in the arterial phase, with centripetal progressive reinforcement in the delayed phase [5]. However, MF-ICC in cirrhotic patients might be hypervascular in the arterial phase, which overlaps with the appearance of typical HCC [17]. Furthermore, about 10–20% of HCCs may show hypoenhancement in the arterial phase and hence mimic MF-ICC [1]. MF-ICC is more prone to confusion with HCC than other types of ICC [17]. Previous works have proposed some promising methods for MF-ICC/HCC classification, but their performance is still not satisfactory [8,9,18]. How to accurately and effectively differentiate MF-ICC from HCC has been a difficult issue in clinical research [15].

It is of interest to consider deep learning as a tool for MF-ICC/HCC classification. We reviewed related references and found no study that applied this method to address the problem. In addition, there still exist some deficiencies that need to be solved in these previous studies [19]. First, some existing deep learning models have poor generalization ability and robustness. The extracted multi-level features could not be well fused, which results in difficulty for the model in discriminating similar feature. Second, the network instability during training also causes the loss of feature information to a large extent, which directly affects the model performance. Third, most studies focus on the characteristics of the lesion area and its size (such as histogram and siamese cross contrast neural network [19]) and pay less attention to the surrounding area; however, for clinical diagnosis, the edge features of MF-ICC/HCC are very important indicators.

To fill in this gap and refine the previous method, a strided feature fusion residual network (SFFNet) model was specifically designed to differentiate MF-ICC from HCC. SFFNet is a new residual model which contains multilayer feature fusion module (MFF), stationary residual block (SRB), and attention mechanism convolutional block attention (CBAM) modules. These modules ensure stronger feature extraction and fusion ability for MF-ICC/HCC MRI classification. In order to eliminate irrelevant background and retain more edge information of the lesions at the same time, we proposed a Semi-SP method for region of interest (ROI) selection. The workflow provided in this paper could provide a new prospective MF-ICC/HCC classification, thus helping inform clinical decision making.

In summary, the main contribution of this paper is as follows:

- We apply a deep learning method to MF-ICC/HCC image classification for the first time and propose a new strided feature fusion model, SFFNet. In SFFNet, two new modules, SF and SRB, are proposed. The SF module is used to capture more multi-dimensional features of MF-ICC/HCC and perform effective fusion, so as to construct more judgment fusion features; the SRB module is used to solve the information loss and network performance instability in the process of residual network training. In addition, the SFFNet model also adds a CBAM attention mechanism to capture key information.
- For T2 weighted imaging (T2WI ) of MF-ICC/HCC, we established a new preprocessing method, Semi-SP, which focuses on the edge information of lesions, which has not received attention in previous studies, and provides qualitative indexes for lesion segmentation.
- The Semi-SP method and SFFNet model proposed in this paper have achieved excellent performance on MR images of 112 MF-ICC/HCC patients in the Second Affiliated Hospital of Kunming Medical University and have obvious advantages in clinical diagnosis compared with other classification models.

The rest of this article is organized as follows: Section 2 introduces the dataset, the theory, and the framework of the Semi-SP method and SFFNet model. Section 3 shows experimental results. Section 4 discusses and analyzes the experimental results. Section 5 summarizes the paper and expands some further research directions.

## 2. Materials and Methods

**Figure 1:**
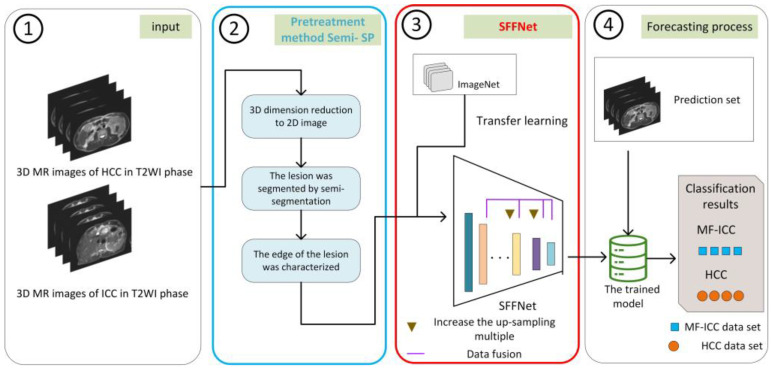
The pipeline of the proposed method.

The MRI classification process of MF-ICC/HCC proposed in this paper is shown in Figure 1, which includes four parts: image input, the data preprocessing method Semi-SP, the SFFNet model, and classification. The novel deep-learning-based workflow, including the SFFNet diagnostic model and the data preprocessing method Semi-SP, are marked in red and blue boxes in Figure 1, respectively.

### 2.1. Patient Selection

Patients with MF-ICC or HCC were retrospectively collected from the Second Affiliated Hospital of Kunming Medical University between July 2015 and September 2021. Inclusion criteria included: (1) underwent a preoperative MRI examination; (2) had no history of treatment for hepatic tumor prior to the study; (3) pathologically confirmed HCC or MF-ICC. Exclusion criteria include: (1) image quality was insufficient for further analysis; (2) T2WI-MRI was incomplete. After screening, we included 47 MF-ICC patients (age range 27–78 years; mean age 58.45 years) with 47 tumor lesions, and 65 HCC patients (age range 31–92 years; mean age 52.68 years) with 69 tumor lesions.

The studies involving human participants were reviewed and approved by the Ethical Committee of the Second Affiliated Hospital of Kunming Medical University. The ethics committee waived the requirement of written informed consent for participation.

### 2.2. MRI Acquisition Protocol

**Figure 2:**
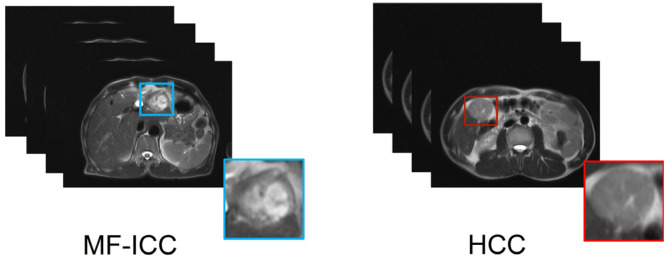
Collected mass-forming intrahepatic cholangiocarcinoma (MF-ICC)/hepatocellular carcinoma (HCC) T2 weighted imaging and corresponding lesion images (left MF-ICC, right HCC).

MRI was performed by a 1.5T MRI scanner (Sonata; Siemens Healthcare; Erlangen, Germany). All images were obtained using half-Fourier acquisition single-shot fast spin-echo sequence, and the scan parameters were as follows: repetition time 1000 ms, echo time 93 ms, slice thickness 8 mm, image matrix 320 × 275, field of view 36 cm × 27 cm, and flip angle 150°. The MRI protocol was 3D T2WI; each 3D sequence image consists of 15–20 slices with different sizes (including 512 × 384 and 512 × 448). Each slice of the 3D image was resized to 224 × 224. Not all the 2D images contained the structural signal of the tumor; we selected 258 and 216 slices of HCC and MF-ICC for further analysis. The T2WI MRI images of two categories are shown in Figure 2.

### 2.3. Image Processing

#### 2.3.1. Tumor Lesion Outlining

Upper abdominal MRI scans (DICOM format) of each patient were imported into the open-source software 3D SLICER (version 5.0.3). Without knowing the pathological grade and clinical information, two radiologists with more than 5 years of experience in abdominal imaging diagnosis outlined the lesion along the tumor boundary layer by layer on a T2WI-MRI lateral scan. If there was any disagreement between the two radiologists, a senior radiologist with 11 years of experience in abdominal imaging diagnosis made the final decision. Then, the outlined images were exported in NRRD format.

#### 2.3.2. Semi-segmented Preprocessing Method and ROI Selection

Presently, there is no uniform standard for the ROI selection; the entire liver or tumor lesion were frequently used in previous studies [14,20]. However, the entire liver contains irrelevant background, and the cirrhotic liver of some HCC patients might influence the classification results. Conversely, only choosing the tumor lesion as the ROI may lose beneficial edge information [16]. Particular care should be taken to avoid too small or too large an ROI; thus, an effective and simple ROI selection criterion is definitely needed.

**Figure 3:**
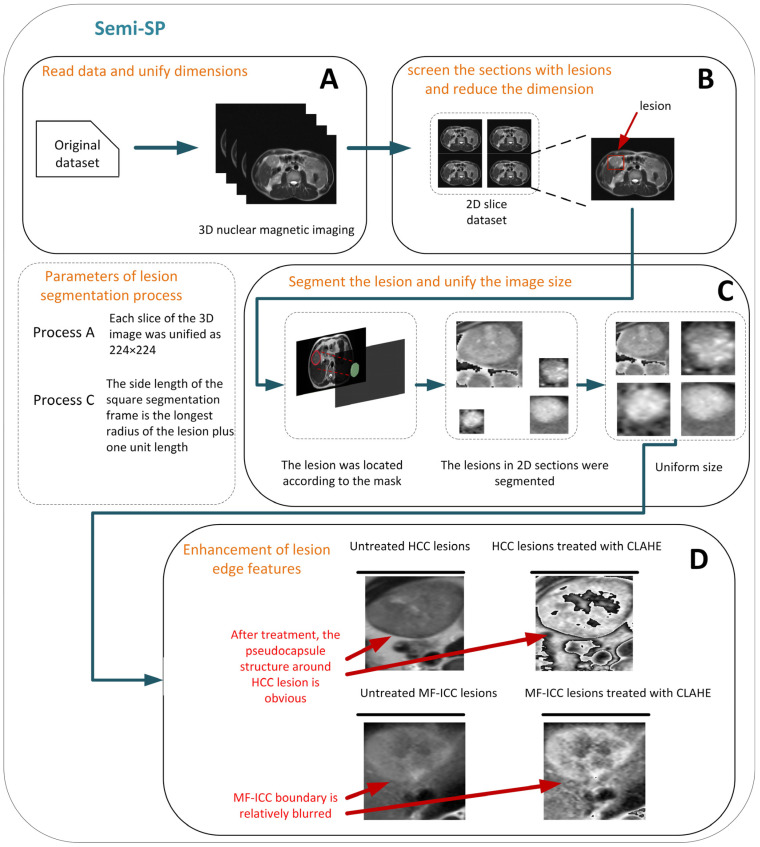
The pipeline of the preprocessing method semi-segmented preprocessing (Semi-SP).

In this study, we proposed a novel semi-segmented preprocessing (Semi-SP) method. The tumor lesion was located according to the boundaries contoured by the radiologists; the maximum radius of the lesion was chosen as the length of the square box to segment the ROI. Namely, the ROI was a square containing the MF-ICC/HCC lesion, and the length of the square depended on the maximum diameter of the lesion. Next, each ROI was scaled to a uniform size of 224 × 224. This scaling method could maintain the feature of the lesions without additional filling pixels. In order to extract more detailed edge information, we highlighted the edge features of HCC/ICC lesions using contrast limited adaptive histogram equalization (CLAHE). The entire pipeline of Semi-SP is shown in Figure 3.

#### 2.3.3. Data Augmentation

Deep learning models require a large amount of data for training. Due to the small number of patients included in this study, data augmentation was applied to reduce the possibility of over-fitting. We utilized geometric transformation (translation, scaling, and rotation) as the method to augment the training and validation set. The number of images in the training and validation set was increased from 474 images (216 for MF-ICC and 258 for HCC) to 2207 images (1206 for HCC and 1001 for MF-ICC). The testing set only applied Semi-SP for segmentation and was not considered for data augmentation.

### 2.4. Deep Learning Model Construction

**Figure 4:**
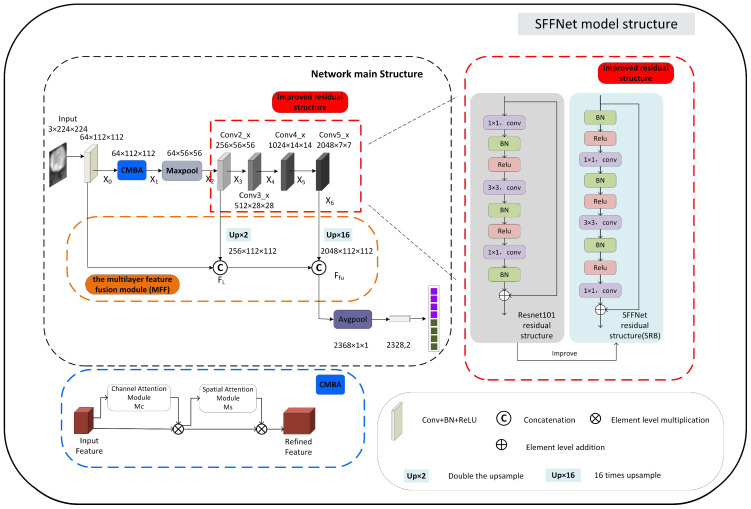
The network structure of strided feature fusion residual network (SFFNet). The multilayer feature fusion module (MFF), stationary residual block(SRB), and convolutional block attention module (CBAM) modules were marked in orange, blue, and red dotted boxes.

In this study, an SFFNet model was specifically designed to differentiate MF-ICC from HCC. First, we chose ResNet101 as the base network model [21]. Transfer learning based on ImageNet was applied for model pre-training. In order to obtain more informative features, the multilayer feature fusion module MFF, the residual structure SRB, and the attention module CBAM were selected to construct the SFFNet model. Details of the SFFNet model are provided in Figure 4.

#### 2.4.1. The Multilayer Feature Fusion Module MFF

**Figure 5:**
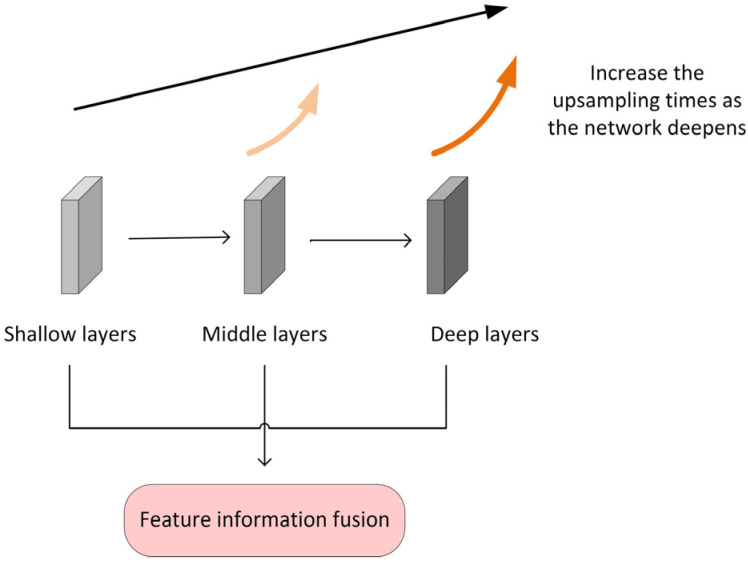
Schematic of the MFF module. With the deepening of the network, the upsampling times are increased to maintain the high resolution of image features.

Low-level features learned in shallow layers retain the spatial information, while the high-level features learned in deep layers contain more semantic information; both low- and high-level features are essential for classification [22]. In SFFNet, the primary role of the MFF module is feature information fusion; thus, it can maintain the high resolution of image features and reduce the information loss in the training process by direct mapping. The schematic of the MFF module is shown in Figure 5.

In order to obtain higher resolution lesion edge texture features, we deal with features acquired from shallow layers, and this process can be expressed by Equations (1) and (2):

(1) $$X_{3}=H(X_{0},W_{l})$$

(2) $$F_{L}=concat(X_{0},up_{2}(X_{3}))$$

where $X_{0}$ and $X_{3}$ are the output vectors of each layer, respectively, $X_{i}\in R^{B\times C\times H\times W}$; (H,W) is the resolution of the image, B is batch size, C is the number of channels, $H(X_{0},W_{1})$ is the mapping to be learned, which contains the attention mechanism, $W_{1}$ is a linear mapping, and ${\mathrm{up}}_{2}(x_{3})$ is twice the upsampling.

Then, the shallow features are fused with the deep features across layers to reshape the feature space. In the process, the deep layer is upsampled by 16 times by bilinear interpolation, which is the same as the process of shallow layers. Still, a significant multiple of upsampling is used to reduce the abstraction of deep layer features and improve the resolution.

(3) $$X_{6}=F(X_{3},W_{2})$$

(4) $$F_{\mathrm{fu}}=concat(F_{L},up_{16}(X_{6}))$$

Similarly, $X_{3}$ and $X_{6}$ are the output vector, $F(X_{3},W_{2})$ is the mapping to be learned, $W_{2}$ is a linear mapping, and ${\mathrm{up}}_{16}(x_{6})$ is 16 times the upsampling.

By modifying the resolution of the feature matrix, the MFF module can better fuse the shallow and deep features so as to achieve the purpose of obtaining the edge features of the lesion.

#### 2.4.2. The Stationary Residual Block SRB

In order to improve the residual structure of ResNet101, in the SFFNet model, a new residual structure SRB was applied.

As shown in Figure 4, the normalized layer BatchNorm2d and the activation function Relu were added before the first convolutional layer with a 1 × 1 convolution kernel of the residual structure. The normalization layer can enhance the ability of network backpropagation and adjust the neural network in time. When errors are found, it can effectively cooperate with the filtering effect of the Relu function and also ensures the correctness of the feature extraction process. In terms of network performance, BatchNorm2d avoided the problem that the network is difficult to train due to different data distributions by unifying the data distribution passed to the Relu function. The Relu function increases the nonlinearity of the neural network, making the network more stable.

Although ResNet101 adopted Relu functions, the generalization ability of this network is weaker. Conversely, in SFFNet, the activation function added to the residual structure decreases the number of “working” neurons, thus increasing the network sparsity and generalization ability. Because conv2_x, conv3_x, conv4_x, and conv5_x are consecutive, it is unnecessary to use continuous normalization layers between them, so the BatchNorm2d function after the third 1 × 1 convolutional layer of the residual structure is removed.

#### 2.4.3. CBAM Attention Mechanism Added

To improve the learning ability of the SFFNet model, we added the attention mechanism CBAM module [23]. It is a highly lightweight attention mechanism, which processes the input data from the two dimensions of space and channel, so that the global relationship of the image is well extracted. Specifically, the channel attention module is in the front, focusing on the meaningful content in the image; the spatial attention module is in the back, focusing on the regions of key pixels. These two parts benefit the imaging diagnosis of MF-ICC/HCC. 

To achieve the full potential of the CBAM attention mechanism, we add the channel and spatial attention structure after the conv1 layer (before the maximum pooling layer Maxpool, after the BatchNorm2d function and Relu function). It should be noted that if the attention mechanism was added to the residual structure, the vital structure of the network would be affected, and the pre-training parameters could not be applied, thus influencing the network learning. Because of this, the attention mechanism and the residual structure are separated in the SFFNet model (see Figure 4).

#### 2.4.4. Data Division

The T2WI measurements of a total of 116 lesions of MF-ICC/HCC patients were randomly divided into the training set (27 MF-ICC and 41 HCC cases), validation set (9 MF-ICC and 14 HCC cases), and testing set (11 MF-ICC and 14 HCC cases) according to the ratio of 6:2:2.

#### 2.4.5. Hyperparameter Optimization

The learning rate decay strategy is adopted to select the optimal learning rate. The initial value was set to 0.001, and the learning rate was updated with a multiplicative factor of 0.1 every 30 epochs. Stochastic Gradient Descent (SGD) was adopted as the optimizer, the CrossEntropyloss function was selected as loss function, the batch size was set to 16, and the epoch was 100. The precision, recall, F1-score, accuracy, and AUC were selected to evaluate the model performance.

All algorithms were implemented in Pytorch framework (version 1.7.0) and run on a 64-bit Windows 10 computer with an Intel(R) Core(TM) i7-10700F CPU running at 64 GB of RAM and an NVIDIA GeForce RTX 3060 GPU with 8GB memory.

## 3. Results

**Figure 6:**
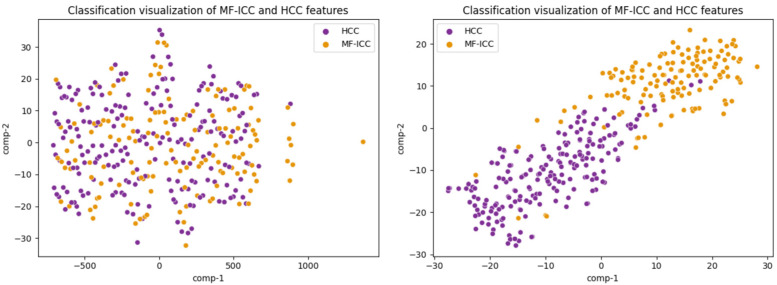
Feature separability of two categories (the left figure shows the original feature separability of the data, and the right figure shows the feature separability of the prediction set after classification by the SFFNet model; purple and yellow dots represent MF-ICC and HCC, respectively).

As shown in Figure 6, the effectiveness of SFFNet is visually verified and evaluated through t-distributed Stochastic Neighbor Embedding (TSNE). This method can verify the grasp of the SFFNet model on the image features of MF-ICC/HCC in an intuitive way. After SFFNet network training, the features of MF-ICC and HCC have high aggregation; thus, the feature separability was increased.

### 3.1. Experimental Results of Different Lesion Segmentation Methods

To prove the effectiveness of the new preprocessing method, we compared Semi-SP with different segmentation strategies. The ROI of the Semi-SP was chosen as the baseline area. Then, the segment area was determined by twice the size of the baseline area, triple the size of the baseline area, the whole image without segmentation, and only the lesion area. As shown in Table 1, the Semi-SP method has the highest precision, recall, F1-score, accuracy, and AUC. In the case which applied the whole image without segmentation, unstable classification results appeared. For MF-ICC/HCC lesions, Semi-SP is more suitable than other segment strategies.

### 3.2. Ablation Experiment Results

The ablation experiment was performed to further validate the performance of the SFFNet model. The ResNet101, ResNet101+CBAM, ResNet101+SRB, ResNet101+MFF, and SFFNet models were compared. All models were pre-trained and under the same parameter settings.

**Figure 7:**
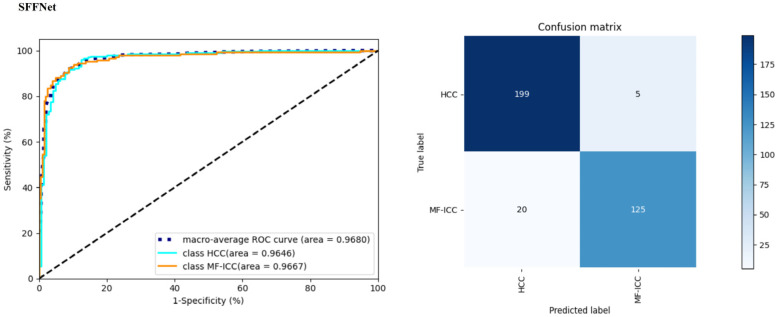
The left is the receiver operating characteristic curve (ROC ) and the right is confusion matrix of the SFFNet model (the blue line indicates the ROC of HCC, the orange line is the ROC of MF-ICC, and the dashed line is the overall ROC).

**Figure 8:**
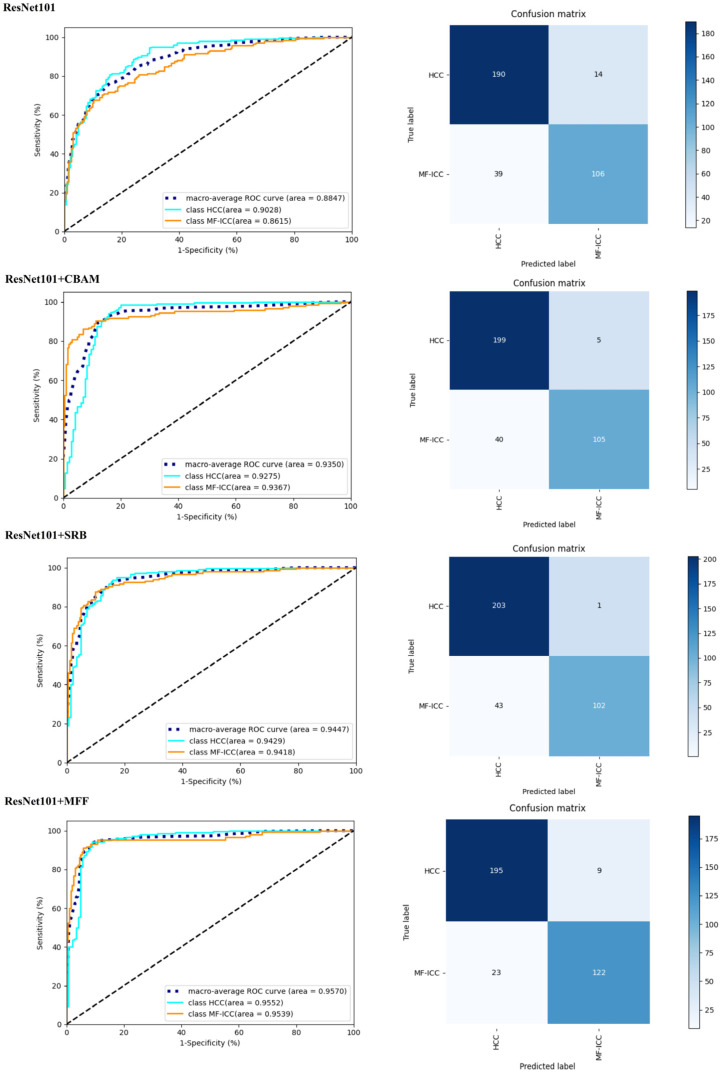
The left is the receiver operating characteristic curve (ROC) and the right is the confusion matrix related to the ablation experiment (the blue line is the ROC of HCC, the orange line is the ROC of MF-ICC, and the dashed line is the overall ROC).

**Figure 9:**
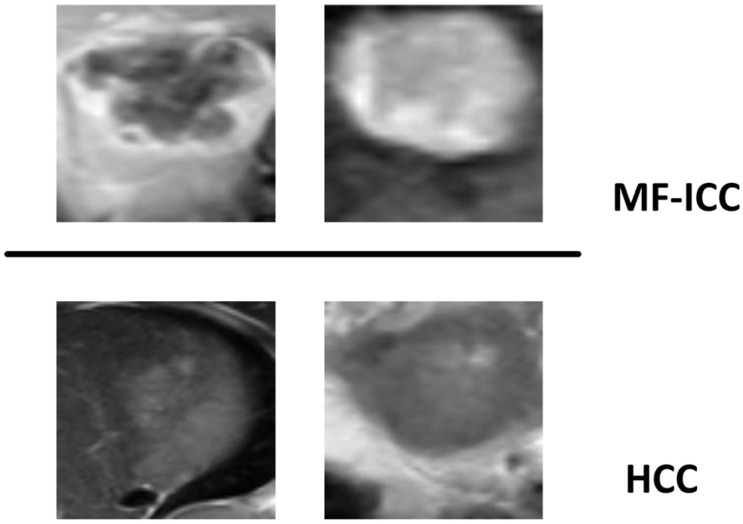
MF-ICC/HCC lesion images incorrectly classified by the SFFNet model in the validation set (top: MF-ICC image was misclassified as HCC; bottom: HCC image was misclassified as MF-ICC).

Table 2 shows the precision, recall, F1-score, accuracy, and AUC values, and Figure 7 and Figure 8 show the ROC curve and confusion matrix of each model. It can be seen that the CBAM, SRB, or MFF module can improve the performance of the ResNet101 model. Among them, MFF was the module that was associated with the largest increase in accuracy and AUC, indicating the importance of the multilayer feature fusion mechanism for feature extraction. Figure 9 shows the MF-ICC/HCC MRI misclassified by the SFFNet model in the prediction set. It can be seen that the boundaries of the misclassified HCC lesion images are relatively blurred, while the boundaries of the MF-ICC lesion images are slightly clearer.

The SFFNet model, which combined CBAM, SRB, or MFF modules, has the highest precision, recall, F1-score, accuracy, and AUC. Comparing the ResNet101 and SFFNet models, the overall accuracy improved by 8.02%, AUC by 8.33%, precision of MF-ICC by 11.12%, F1-score by 9.96%, and other indicators increased by more than 6%. In addition, the SFFNet model has high sensitivity for identifying both types of tumor, thus proving the applicability of the SFFNet model for MF-ICC/HCC classification.

### 3.3. Model Comparison

**Figure 10:**
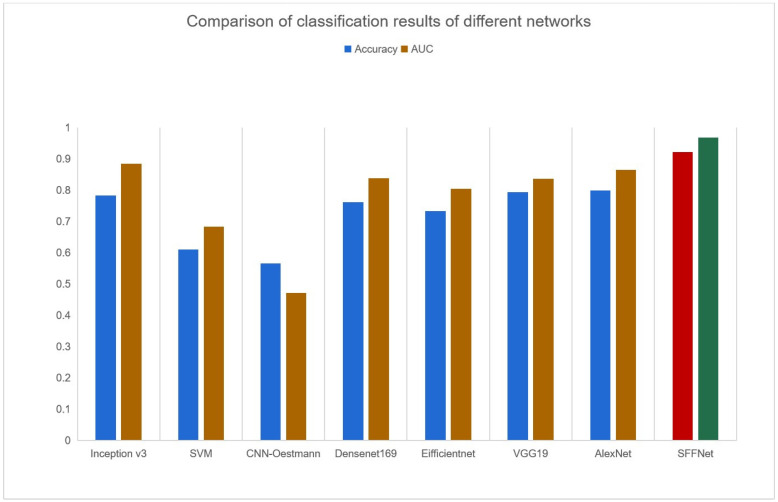
The experimental results of different networks (yellow and blue represent the classification accuracy and AUC value of each model, while red and green highlight the accuracy and AUC value of the model SFFNet).

As shown in Table 3, we compared the SFFNet with other widely used classification methods, including Densenet169, Eifficientnet, VGG19, and AlexNet, as well as models used in previous studies, including SVM [20], Inception v3 [24], and a 3D CNN model used applied by Oestmann et al. (CNN-Oestmann) [14]. Figure 10 shows the histogram visualization of eight models. SFFNet has better performance than the other seven models, indicating the methods proposed in this study might have better applicability for MF-ICC/HCC T2WI image classification.

## 4. Discussion

In this paper, we proposed a specifically designed Semi-SP method and SFFNet model to differentiate MF-ICC from HCC. The model achieved an overall accuracy of 92.26% and an AUC of 0.9680, with high sensitivity (86.21%) and specificity (94.70%) for MF-ICC. The proposed workflow had better performance than other widely used classification methods in previous studies, which is an encouraging result achieved in MF-ICC/HCC classification.

As shown in Figure 3, HCC lesions have clear boundaries and pseudocapsule structures, while the boundaries of MF-ICC are relatively blurred. The features of the lesion edge are essential for MF-ICC/HCC classification, but the irrelevant background might “mislead” the classification models. Thus, the crucial issue is how to reduce the redundant features and retain the key edge information. Different segment strategies could have an unneglectable effect on the classification results. 

In this study, we proposed a Semi-SP preprocessing method. By enlarging the contrast features of MF-ICC/HCC lesions, it can identify the presence of a pseudocapsule or bile duct dilatation. Semi-SP achieved better performance compared to other segment strategies, so this method might be more suitable for MF-ICC/HCC classification.

For feature extraction, we introduced the MFF module to ensure stronger feature extraction. As shown in the ablation experiment results, this module brings the best performance increase in the ablation experiment. This indicate that morphological information of the lesions (low-level features learned in shallow layers) can greatly improve the accuracy of model classification. Fusing low-level features with high-level features could strengthen the iteration of pixel and semantic information and provide more effective features for MF-ICC/HCC classification. 

Since we reduce the receptive field of the model during the training process, the CBAM attention mechanism was applied to enhance the learning ability of the network model. By focusing on each part of the images, CBAM can identify the crucial information and suppress the redundant features. The ablation experiment also verified the auxiliary role of this module.

For the residual network, with the continuous increase in the number of network layers, the fluctuation of the signal transmitted also increases accordingly, resulting in problems such as difficulty in network training [17]. In addition, the sampling process might cause information loss and unstable network performance [19]. Considering these defects, a normalization layer was adopted in this study to prevent overfitting and improve the generalization ability. With the addition of the activation function, the sparsity of the network was improved and the interdependence between parameters was reduced. These improvements could solve the problem of increasing signal fluctuation and decreasing feature expression of the network, thus enhancing the expression ability of the network.

In this study, we also compared SFFNet with seven other widely used classification methods. As shown in Table 3, the classification performance of T2WI images of MF-ICC/HCC by SFFNet was better than other models. Compared with the previous study, we included more previously ignored edge information. From the misclassified results, we can find that the misclassified HCC lesion had a similar surrounding area to MF-ICC. As shown in Table 1, when the boundary information of some lesions is taken into account and interference information in the environment is removed, the classification accuracy is significantly improved, which indicates that the edge region may have key information for classification, which is an important inspiration and reminder for subsequent MF-ICC/HCC classification studies. In addition, from the perspective of the model, the SF module, SRB module, and CBAM module in SFFNet are helpful for improving the accuracy. It can be seen that the SFFNet model is more suitable for MF-ICC/HCC MRI classification. 

## 5. Conclusions

In this study, an effective new model, SFFNet, and preprocessing method, Semi-SP, were established for the imaging diagnosis of MF-ICC/HCC, which filled the gap in the deep learning field of MF-ICC/HCC diagnosis. Based on the obtained results, the following conclusions can be drawn: 

1. The Semi-SP preprocessing method proposed in this paper focuses on the defects of previous studies, better retains the edge image of the lesion, and constructs a higher-resolution lesion image. 

2. In the SFFNet model, we propose the stride feature fusion module SF to broaden the feature extraction space and effectively fuse multiple features to form more judgment fusion features. The SF module enables the model to identify more lesion features; a new residual block SRB is also proposed to solve the problem of feature information loss during training, so as to improve the robustness of the residual network and ensure that more lesion features can be preserved. At the same time, we also use the attention mechanism CBAM to assist the model to extract key information and reduce the intake of irrelevant information, so as to ensure that the model can focus on the key areas of the lesion.

3. In the end, the workflow achieved an overall accuracy of 92.26% and an AUC of 0.9680, with high sensitivity (86.21%) and specificity (94.70%) for MF-ICC. Good classification performance indicates that this workflow could provide a new prospective MF-ICC/HCC classification method and provide complementary information for personalized treatment strategy. 

4. In view of the importance of multi-parameter MRI in the early diagnosis of MF-ICC/HCC, we will collect other MRI, such as delayed phase and arterial phase, and try to fuse multi-parameter MRI to assist diagnosis to achieve better classification performance.

## Figures and Tables

**Table 1:** Comparative experiments of different preprocessing methods.

Preprocessing Method	Classification Type	Precision	Recall	F1-Score	Accuracy	AUC
Semi-SP	HCC	0.9078	0.9657	0.9359	0.9226	0.9680
	MF-ICC	0.9470	0.8621	0.9025		
Split size doubled	HCC	0.8246	0.8529	0.8386	0.8080	0.8930
	MF-ICC	0.7826	0.7448	0.7633		
Split size tripled	HCC	0.6368	0.8828	0.7399	0.7421	0.8477
	MF-ICC	0.4179	1.0000	0.5894		
Do not segment the lesion	HCC	1.0000	0.0098	0.0194	0.4212	0.6284
	MF-ICC	0.4179	1.0000	0.5894		
Only segment lesions	HCC	0.9150	0.8971	0.9059	0.8911	0.9436
	MF-ICC	0.8591	0.8828	0.8707		

Abbreviation: F1-Score: F1 Measure; Semi-SP: semi-segmented preprocessing; AUC: Area Under the Curve; MF-ICC: mass-forming intrahepatic cholangiocarcinoma.

**Table 2:** Ablation experiments.

Network Model	Classification Type	Precision	Recall	F1-Score	Accuracy	AUC
ResNet101	HCC	0.8465	0.8922	0.8687	0.8424	0.8847
	MF-ICC	0.8358	0.7724	0.8029		
ResNet101+CBAM	HCC	0.8916	0.8873	0.8894	0.8711	0.9350
	MF-ICC	0.8425	0.8483	0.8454		
ResNet101+SRB	HCC	0.9050	0.8873	0.8960	0.8797	0.9447
	MF-ICC	0.8456	0.8690	0.8571		
ResNet101+MFF	HCC	0.8739	0.9853	0.9263	0.9083	0.9570
	MF-ICC	0.9748	0.8000	0.8788		
SFFNet	HCC	0.9078	0.9657	0.9359	0.9226	0.9680
	MF-ICC	0.9470	0.8621	0.9025		

**Table 3:** Comparison of different classification models.

Classification Model	Classification Type	Precision	Recall	F1_score	Accuracy	AUC
SVM	HCC	0.5909	0.8667	0.7027	0.6099	0.6834
	MF-ICC	0.6774	0.3182	0.4330		
CNN-Oestmann	HCC	0.5455	0.8000	0.6486	0.5667	0.4717
	MF-ICC	0.6250	0.3333	0.4348		
Inception v3	HCC	0.8333	0.7843	0.8081	0.7822	0.8845
	MF-ICC	0.7197	0.7793	0.7483		
Densenet169	HCC	0.8363	0.7402	0.7844	0.7622	0.8388
	MF-ICC	0.6845	0.7931	0.7348		
Eifficientnet	HCC	0.8171	0.7010	0.7546	0.7335	0.8035
	MF-ICC	0.6494	0.7793	0.7085		
VGG19	HCC	0.7946	0.8725	0.8318	0.7936	0.8364
	MF-ICC	0.7920	0.6828	0.7333		
AlexNet	HCC	0.8190	0.8431	0.8309	0.7994	0.8657
	MF-ICC	0.7698	0.7379	0.7535		
SFFNet	HCC	0.9078	0.9657	0.9359	0.9226	0.9680
	MF-ICC	0.9470	0.8621	0.9025		

## Data Availability

The data that support the findings of this study are available from the corresponding author upon reasonable request.
